# Osteosarcoma: Current Concepts and Evolutions in Management Principles

**DOI:** 10.3390/jcm12082785

**Published:** 2023-04-09

**Authors:** Pampina Pilavaki, Amir Gahanbani Ardakani, Panagiotis Gikas, Anastasia Constantinidou

**Affiliations:** 1 Medical School, University of Cyprus, Nicosia 1678, Cyprus; 2 Medical Oncology, Bank of Cyprus Oncology Center, Nicosia 2006, Cyprus; 3 Department of Orthopaedics, Cleveland Clinic London, London SW1X 7HY, UK; amir.ardakani1@nhs.net; 4 Cyprus Cancer Research Institute, Nicosia 2109, Cyprus

**Keywords:** osteosarcoma, chemotherapy, targeted therapy, immunotherapy, immune checkpoint inhibitors, adoptive cellular therapy limb salvage therapy

## Abstract

Osteosarcoma is a rare malignancy arising from mesenchymal tissue, and represents the most common bone sarcoma. The management of osteosarcoma is challenging, and requires a multidisciplinary approach. In daily clinical practice, surgery, radiotherapy, and conventional chemotherapy constitute the therapeutic armamentarium against the disease. However, a significant number of patients with initially localized osteosarcoma will experience local or distant recurrence, and the prognosis for metastatic disease remains dismal. There is a pressing need to identify novel therapeutic strategies to better manage osteosarcoma and improve survival outcomes. In this study, we present recent advances in the therapeutic management of osteosarcoma, including surgical and medical advances. The role of immunotherapy (immune checkpoint inhibitors, adoptive cellular therapy, cancer vaccines) and other targeted therapies including tyrosine kinase inhibitors is discussed; however, additional studies are required to delineate their roles in clinical practice.

## 1. Introduction

Osteosarcoma is a rare malignancy of mesenchymal origins, which is characterized by the production of osteoid from the neoplastic cells [1,2]. It is the most common bone sarcoma, with an estimated incidence in Europe of 0.3/100,000/year [3]. The incidence of osteosarcoma demonstrates a bimodal age distribution, with an initial peak in adolescence (0.8–1.1/100,000/year in the age group of 15–19 years) that coincides with the pubertal growth spurt; a second peak in the seventh and eighth decades of life often represents a secondary malignancy, or is related to Paget disease [3,4]. Approximately two-thirds of the primary tumors are located around the knee joint, with the most common locations being the distal femur, the proximal tibia, and the proximal humerus [5,6].

The diagnosis of osteosarcoma relies on morphological characteristics, since specific molecular testing is not yet available in clinical practice [3]. According to the latest World Health Organization (WHO) classification, high grade osteosarcoma subtypes include conventional osteosarcoma, which is the most common subtype, telangiectatic osteosarcoma, and small cell osteosarcoma [7]. Periosteal osteosarcoma, which is predominantly chondroblastic, is an intermediate-grade osteosarcoma, whilst low-grade central osteosarcoma and parosteal osteosarcoma are reported as low-grade neoplasms.

Both the diagnosis and management of osteosarcoma are challenging, and require a multidisciplinary approach. Currently, surgical excision, radiotherapy, and multiagent systematic therapy constitute the armamentarium of therapies against osteosarcoma in daily clinical practice [3]. However, it has been reported that 30–40% of patients with local osteosarcoma will eventually experience local or distant recurrence, with the 5-year overall survival for recurrent disease being 23–29% [8,9]. Furthermore, 10–20% of patients originally present with macroscopic metastatic disease, whilst the lungs are the most common site of metastases [5,8].

Given the high rate of relapse and the poor prognosis of metastatic disease, there is a pressing need to identify novel therapeutic approaches and biomarkers to better manage the disease. In the era of precision medicine, several efforts have been made to better understand the complex biology, genetic, and molecular background of osteosarcoma, and to develop new targeted therapies [6].

## 2. Current Therapeutic Approaches

The standard of care for localized low-grade osteosarcoma, such as low-grade central osteosarcoma and parosteal osteosarcoma, involves the surgical excision of the tumor with clear margins (R0 resection) [3]. As for localized periosteal osteosarcomas, neoadjuvant or adjuvant chemotherapy is not recommended, since there is no solid evidence of benefit from its use [3,10]. In contrast, for localized high-grade osteosarcoma, curative intent treatment requires neoadjuvant multiagent chemotherapy, followed by surgical excision and adjuvant chemotherapy. The incorporation of chemotherapy for the therapeutic management of localized high-grade osteosarcoma was established in the 1980s, and has improved the disease-free survival probability from <20% to >60% [3,6]. First-line multiagent chemotherapy comprises doxorubicin, cisplatin, and high-dose methotrexate (MAP regimen) [3]. The histological response to neo-adjuvant therapy with MAP is evaluated post-surgery; however, changing the chemotherapy regimen based on this information has not been proven to improve outcomes [11,12]. Specifically, EURAMOS-1, a phase III, open-label, randomized, controlled clinical trial, investigated the addition of pegylated interferon alfa-2b (IFN-a-2b) to the MAP regimen in patients who responded to neo-adjuvant therapy (<10% viable tumor); meanwhile, in poor responders (≥10% viable tumor), the addition of ifosfamide and etoposide to MAP (MAPIE) was investigated. The results showed no improvement in event-free survival (primary end point) compared to patients who were treated with adjuvant MAP [11,12]. Furthermore, radiotherapy is indicated in selected cases where surgical resection of the tumor is not feasible, or the risk of local recurrence is high and additional surgery cannot be applied [3].

For many years, therapy in cases of unresectable metastatic or recurrent osteosarcoma relied on chemotherapy, and included ifosfamide or cyclophosphamide which is given with etoposide and/or carboplatin [3]. Moreover, a combination of gemcitabine and docetaxel has been used as further line therapy. In addition, radiotherapy may have a role in the palliative setting for symptom control, mainly pain [3]. Importantly, for patients with primary metastatic disease, the same principles as those applied in localized osteosarcoma are followed [3]. In cases of local recurrence as the first event, the treatment is primarily surgical, with no evidence of benefits from chemotherapy [3,13]. Regarding cases with recurrent osteosarcoma with isolated lung metastasis, metastasectomies are considered to be the optimal treatment [3]. However, stereotactic radiotherapy, radiofrequency ablation, or cryotherapy can be used as alternatives if patients are unfit for surgery [3].

## 3. Advances in Targeted Therapies for Osteosarcoma

### 3.1. Clinical Experience of Immunotherapy Approaches in Osteosarcoma

Immunotherapy, a rapidly evolving area in oncology, has been applied with success in several malignancies including melanoma [14], non-small cell lung cancer [15,16], and renal cell carcinoma [17]. Novel immunotherapy approaches, including immunomodulating antibodies, adoptive cellular therapy, and cancer vaccines, have been investigated in several clinical studies that enrolled patients with different types of sarcomas, including osteosarcoma [18,19,20,21]. Selected clinical studies which have evaluated different types of immunotherapies in patients with osteosarcoma are presented in Table 1.

**Immune checkpoint inhibitors** have been evaluated in patients with osteosarcoma, as a single agent therapy or in combination with chemotherapy, with disappointing results [22,23,24]. The SARC028, a phase 2, non-randomized, open-label, single-arm, multicenter, two-cohort clinical trial, investigated the single agent pembrolizumab (200 mg IV every 3 weeks) in patients with advanced soft tissue sarcoma and bone sarcoma [22]. With regards the bone sarcoma arm, 40 patients were evaluable for response, 22 of which were diagnosed with osteosarcomas, 13 with Ewing sarcoma, and 5 with chondrosarcoma. The primary end point was the objective response rate (ORR) by RECIST 1.1 criteria, which was 5% for bone sarcoma with a 43-week median duration of response. Among the patients who were diagnosed with osteosarcomas, 1 experienced a partial response (PR), 6 had stable disease (SD), and 15 experienced progression of the disease (PD). The median progression-free survival (PFS) and the median overall survival (OS) for the bone sarcoma arm were 8 weeks (95% CI 7–9) and 52 weeks (95% CI 40–72), respectively.

Combinations of immune checkpoint inhibitors with chemotherapy have been investigated in patients with osteosarcomas as well. The PEMBROSARC clinical trial, a phase 2, multicenter, open-label study, explored a combination of pembrolizumab (200 mg IV every three weeks) with cyclophosphamide (50 mg twice daily; one week on followed by one week off) [23]. Seventeen patients with metastatic and/or unresectable osteosarcomas were enrolled in one of its strata, 15 of which were evaluable for efficacy. The primary end points were determined as the non-progression and objective responses at 6 months, as per the RECIST 1.1 criteria. Specifically, the 6-month non-progression rate was 13.3%. The results showed that one patient (6.7%) experienced PR, 5 patients (33.3%) had SD, and 8 patients (53.3%) had PD. The median PFS and the median OS were 1.4 months (95% CI = 1.0 months–1.4 months) and 5.6 months (95% CI = 2.1 months–12.1 months), respectively. Additionally, 14 patients were tested for PD-L1 expression, and only 2 were positive; interestingly, the one who experienced a PR did not express PD-L1.

**Adoptive cellular therapy,** an innovative immunotherapeutic approach, has been evaluated in osteosarcomas, with encouraging results. In 2020, Shi J. et al. published the results of a retrospective study that explored the efficacy of adjuvant chemotherapy in combination with tumor-infiltrating lymphocytes (TILs) treatment in patients with primary high-grade intramedullary osteosarcoma who had previously received neoadjuvant chemotherapy and had poor response, as proven histologically [25]. In total, 80 patients were included in the study; 40 patients (group 1) were treated with adjuvant chemotherapy, and 40 patients (group 2) received adjuvant chemotherapy in combination with TILs therapy. In both groups, neoadjuvant and adjuvant chemotherapy comprised the MAP regimen. The median disease-free survival (DFS) and median OS were estimated for both groups. For group 1, the median DFS was 55.5 months, and the median OS was 80.4 months; for group 2, the median DFS was 65.3 months and the median OS was 95.8 months. Several factors were examined as potential prognostic factors; interestingly, a greater number of transfused TILs were proposed as a potential prognostic factor, which is associated with increased median PFS and OS in osteosarcomas treated with adjuvant chemotherapy and TILs therapy. Additionally, PD1 expression by CD3^+^CD8^+^TILs from tumor specimens was examined, and the association between CD3^+^CD8^+^PD1^+^TILs and the prognosis of osteosarcoma was analyzed. No significant difference was reported between the two groups regarding the PD1 expression on CD3^+^CD8^+^TILs. The patients in group 1 and group 2 were further divided into PD1^hi^ (≥10%) and PD1^low^ (<10%), based on the expression of PD1 on CD3^+^CD8^+^TILs. Importantly, the results showed that PD1^hi^ was a good prognostic factor for group 2, but appeared to be a poor prognostic factor in group 1. Regarding adverse effects, no significant differences were reported between the two groups.

Further to the above, a combination of TILs therapy with anti-PD1 therapy in patients with metastatic osteosarcoma was investigated through two retrospective studies published in 2020, and showed promising results. Sixty patients with chemotherapy-resistant metastatic osteosarcomas were included in the first retrospective analysis presented by Zhou X. et al. [26]. The patients were administered TILs therapy, with an average of 5 × 10^9^ cells (range, 3–8 × 10^9^) per infusion, combined with nivolumab (3 mg/kg/cycle). The TILs were transfused in the first cycle of nivolumab. Regarding treatment-related adverse effects, two patients experienced grade 3 or 4 toxicity, whilst fever, fatigue, rash, anorexia, leukopenia, and anemia were the most common adverse effects. The ORR and the disease control rate were 36.67% and 80%, respectively. Specifically, 2 patients experienced complete response (CR), and 20 patients had PR. Additionally, the median PFS was 5.75 months, and the median OS was 13.6 months. Of note, more infusions of TIL and CD8+ TIL, fewer infusions of CD8+ PD1+ TIL, and fewer infusions of CD4+ FoxP3+ TIL demonstrated improved PFS and OS. The second retrospective study, presented by Wang C. et al., investigated the efficacy of the combined treatment with anti-PD1 therapy and TILs therapy in metastatic osteosarcoma as well [27]. In total, 60 patients were evaluated in the study; 30 patients (group 1) were treated with nivolumab only (3 mg/kg/cycle, maximum dose of 240 mg/cycle), and 30 patients (group 2) received a combined treatment with TILs, with an average of 5.1 × 10^9^ cells per infusion (range, 3.2–8.9 × 10^9^), and nivolumab (same dose with group 1). In group 1, the ORR was 6.67%, the median PFS was 3.8 months, and the median OS was 6.6 months compared to group 2, in which the ORR was 33.3%, and the median PFS and median OS were 5.4 months and 15.2 months, respectively. Importantly, increased PFS and OS were observed in patients with more infusions of TIL numbers and CD8+ TILs, or fewer infusions of CD8+ PD+ TILs, or fewer infusions of CD4+ FoxP3+ TILs.

Another clinical trial phase 1/2, reported by Ahmed N. et al., which enrolled patients with human epidermal growth factor receptor 2 (HER2) positive sarcomas, explored the use of HER2-specific chimeric antigen receptor (CAR) T cells [28]. In total, 19 patients were included in the study, 16 of which were diagnosed with osteosarcoma. The HER2-CAR T cells were administered in escalating doses (from 1 × 10^4^/m^2^ to 1 × 10^8^/m^2^), and no dose-limiting toxicity was observed. However, the clinical benefit was limited, with the median OS being 10.3 months (range, 5.1–29.1 months).

With regards to **cancer vaccines**, a few clinical studies showed discouraging results for patients with osteosarcoma thus far [29,30]. Himoudi N. et al. conducted a phase 1 clinical trial that investigated the use of dendritic cells (DCs) pulsed with autologous tumor lysate in patients diagnosed with sarcoma [30]. Of the 16 patients included in the study, 13 were diagnosed with osteosarcoma (12 finally received the vaccination), 1 patient was diagnosed with Ewing sarcoma, 1 with medulloblastoma, and 1 with neuroblastoma. The participants were treated with vaccination with autologous DCs matured with autologous tumor lysate and keyhole limpet hemocyanin. Overall, there was no significant toxicity, but there was no significant evidence of clinical benefit either, since only 2 out 12 osteosarcoma patients had a significant anti-tumor response.

Overall, treatment with immune checkpoint inhibitors as a single therapy or in combination with chemotherapy appears to be of limited value in clinical practice [22,23]. It has been suggested that cytotoxic T lymphocytes that are produced during the process of progression of osteosarcoma are exhausted in the tumor. Since the response to PD-1 inhibitors may depend on the number of TILs in the tumor microenvironment, the treatment with PD-1 inhibitors solely may not be efficacious enough for osteosarcomas [26,27]. Furthermore, the investigation of PD-1 inhibitors in combination with modules that could modify the tumor microenvironment has been proposed [23,31]. Thus far, the investigation of anti-PD1 therapy in combination with TILs therapy in patients with metastatic osteosarcoma is a promising therapeutic approach, as it appears to be both safe and effective; however, further research is required to validate the results of the aforementioned retrospective studies [26,27]. Additionally, a combination of adjuvant chemotherapy with TILs may prolong survival in patients with osteosarcoma who have responded poorly to neoadjuvant chemotherapy [25].

**Table 1:** Clinical experience of immunotherapy in patients with osteosarcoma (selected studies).

Immunotherapy Intervention		Type of Study	Number of Patients	Best Response	Survival
**ICI**	Pembrolizumab [22]	Phase 2	22	-PR: 5% (1 patient) -SD: 27% (6 patients) -PD: 68% (15 patients)	-mPFS ^1^: 8 weeks -mOS ^1^: 52 weeks
	Pembrolizumab and Metronomic cyclophosphamide [23]	Phase 2	17	-PR: 6.7% (1 patient) -SD: 33.3% (5 patients) -PD: 53.3% (8 patients)	-mPFS: 1.4 months -mOS: 5.6 months
	Bempegaldesleukin and Nivolumab [24]	Pilot study	10	-PR: 0/10	-mPFS: 2 months -mOS: 6.3 months
**ACT**	Adjuvant chemotherapy ± TILs therapy [25]	Retrospective study	80		(a) Group 1 (adjuvant chemotherapy—MAP regimen): -mDFS: 55.5 months -mOS: 80.4 months (b) Group 2 (adjuvant chemotherapy and TILs therapy): -mDFS: 65.3 months -mOS: 95.8 months
	TILs therapy and anti-PD1 therapy (nivolumab) [26]	Retrospective study	60	-ORR: 36.67% (22 patients: 2 patients CR, 20 patients PR)	-mPFS: 5.75 months -mOS: 13.6 months
	Anti-PD1 therapy (nivolumab) ± TILs therapy [27]	Retrospective study	60	(a) Group 1 (anti-PD1 therapy): -ORR: 6.67% (b) Group 2 (anti-PD1 therapy & TILs therapy): -ORR: 33.3%	(a) Group 1 (anti-PD1 therapy): -mPFS: 3.8 months -mOS: 6.6 months (b) Group 2 (anti-PD1 therapy and TILs therapy): -mPFS: 5.4 months -mOS: 15.2 months
	HER2-specific CAR T cell [28]	Phase 1/2	16	PR: 1 patient ^2^ SD: 3 patients PD: 10 patients NE: 2 patients	-mOS ^3^: 10.3 months
**Vaccines**	Autologous DCs matured with autologous tumor lysate and KLH [30]	Phase 1	13	No clinical response	

ICI, immune checkpoint inhibitors; ACT, adoptive cellular therapy; TILs, tumor-infiltrating lymphocytes; CR, complete response; PR, partial response; SD, stable disease; PD, progression disease; NE, not evaluable; mPFS, median progression-free survival; mOS, median overall survival; mDFS, median disease-free survival; ORR, objective response rate. ^1^ mPFS and mOS for this trial were estimated for the bone sarcoma cohort which included 40 patients (22 osteosarcomas, 13 Ewing sarcomas, 5 chondrosarcomas). ^2^ The patient experienced PD after the first dose of T cells; therefore, he proceeded with salvage chemotherapy followed by a second dose of T cells. A PR lasting for 9 months was observed after the second infusion. ^3^ The mOS for this trial was estimated for all the patients included in the study (19 patients) comprising 16 osteosarcomas, 1 Ewing sarcoma, 1 primitive neuroectodermal tumor, 1 desmoplastic small round cell tumor.

### 3.2. Clinical Experience of Antibody-Drug Conjucates in Osteosarcomas

An antibody–drug conjugate (ADC) is an antibody that targets a specific cell surface protein linked with a cytotoxic agent [6,32]. ADCs are a promising class of therapeutics, especially for tumors which lack oncogenic ‘driver’ pathways [32]. Proteins that are overexpressed on osteosarcoma cells, such as the glycoprotein non-metastatic B (GPNMB), the leucine-rich repeat-containing 15 (LRRC15), B7-H3, GD2, and HER2, could represent potential targets for ADCs [6]. However, the clinical application of this therapeutic approach remains limited, and the results are conflicting.

In 2019, Kopp L.M. et al. reported a single-arm phase 2 clinical trial which explored the anti-tumor activity of glembatumumab vedotin (an ADC against GPNMB) in patients with recurrent or refractory osteosarcoma [33]. Twenty-two adolescents and young adults were enrolled in the study, and were treated with a 1.9 mg/kg/dose of glembatumumab vedotin on day 1 of a 21-day cycle. Disease control at 4 months and RECIST response was the primary end point. Limited clinical activity of glembatumumab vedotin in osteosarcoma patients was demonstrated. Specifically, 1 patient had a PR (4.5%), and 2 SD (9.1%). No correlation between GPNMB expression and response to glembatumumab vedotin was reported. Regarding toxicity, rash and hypokalemia was the most common grade 3 adverse event, and one death from end organ failure was reported that was possibly related to the drug.

In 2021, Demetri G.D. et al. Published the results of a phase 1 clinical trial exploring the safety, pharmacokinetics/pharmacodynamics, and preliminary antitumor activity of ABBV-085 (ADC against LRRC15) in patients with sarcomas and other advanced solid tumors [34]. The recommended expansion dose was determined at 3.6 mg/kg every 14 days. Ten patients with osteosarcomas were included in the trial and treated with 3.6 mg/kg ABBV-085, of which 2 experienced a PR (20%), 2 had SD (20%), and 6 had PD (60%). The most common adverse effects included fatigue, nausea, and decreased appetite. ABBV-085 was safe and tolerable at 3.6 mg/kg every 14 days, and preliminary anti-neoplastic activity was demonstrated in patients with osteosarcoma, suggesting further investigation of this agent is warranted.

### 3.3. Clinical Experience of Tyrosine Kinase Inhibitors (TKIs) and beyond in Osteosarcomas

In recent years, efforts have been made towards the identification of driving molecular and genetic alterations in osteosarcomas, which could be used as potential targets for the management of osteosarcoma [35]. Multi-target TKIs have been tested in several clinical trials that included patients with osteosarcomas. A few TKIs, including sorafenib, regorafenib, cabozantinib, and apatinib, demonstrated promising results, encouraging their further investigation as single therapies or in combination with other agents; their toxicity remains a concern in this case. However, it is still unclear which TKI targets demonstrate a key role for osteosarcoma therapy [36].

**Sorafenib**, a multi-target TKI, was the first TKI to demonstrate clinical activity in osteosarcomas in multicenter prospective clinical trials [36,37,38]. In 2011, Grignani G. et al. presented the results of a phase 2 clinical trial in patients with relapsed and unresectable high-grade osteosarcomas treated with sorafenib (400 mg twice daily) after failure of standard multiagent treatment [37]. In total, 35 patients were enrolled in the study. The PFS at 4 months, which was the primary end point, was 46% (95% CI 28% to 63%). The median PFS was 4 months, and the median OS was 7 months. Three patients (8%) experienced PR, 2 (6%) had minor responses (<30% tumor shrinkage), and 12 (34%) had SD. Importantly, PR/SD lasted ≥6 months in 6 patients (17%). A reduction or brief interruption of sorafenib was reported in 16 patients (46%), and discontinuation in 1 patient (3%) due to toxicity. These results encourage further investigation of TKIs in osteosarcomas.

**Regorafenib** is a multi-target TKI approved for the treatment of advanced or metastatic colorectal cancer, GIST, and hepatocellular carcinoma [36,39,40,41]. At least two clinical trials investigated regorafenib in patients with advanced or metastatic osteosarcomas, and both showed efficacy [42,43]. REGOBONE, a non-comparative, double-blind, placebo-controlled phase 2 trial, evaluated the efficacy and safety of regorafenib in patients with bone sarcomas [42]. In 2018, Duffaud F. et al. reported the results of the osteosarcoma cohort. Forty-three patients were enrolled, 38 of which were evaluable for efficacy. The patients were randomly assigned (2:1 ratio) to regorafenib (26 evaluable patients) or to the placebo (12 evaluable patients). In the regorafenib group, 17 of the 26 patients did not experience progression at 8 weeks (primary end point), whilst no patients were non-progressive at 8 weeks in the placebo group. Two patients with a PR (8%) were observed in the regorafenib group, and none in the placebo group. The median PFSs were 16.4 weeks and 4.1 weeks in the regorafenib and placebo groups, respectively. Seven of the twenty-nine patients in the regorafenib group experienced 13 treatment-related serious adverse events, and no treatment-related deaths were reported. Additionally, Davis L.E. et al. reported the results of another multicenter, randomized placebo-controlled, double-blind, phase 2 clinical trial in patients with metastatic osteosarcomas who received at least one prior line of treatment [43]. The patients were randomly assigned to regorafenib or placebo (1:1 ratio), and crossover was permitted at the time of progression. Forty-two patients were enrolled in the study, and ten patients who received the placebo crossed over to regorafenib once they had progression of disease. Of note, the enrolment of patients was stopped early following a review by the data safety monitoring committee. The median PFS, as the primary end point, was 3.6 months for regorafenib and 1.7 months for the placebo. Moreover, three patients (13.6%) randomly assigned to regorafenib experienced a PR as per RECIST 1.1. Regarding toxicity, however, 14 patients (64%) of the 22 who were initially assigned to regorafenib had grade 3–4 adverse events related to the therapy. Both studies showed meaningful clinical activity of regorafenib in advanced osteosarcomas, which warrants further investigation as a single therapy or in combination with other treatment modalities.

The activity of **cabozantinib** in patients with advanced Ewing sarcoma and osteosarcoma was investigated through the CABONE trial, a multicenter, single arm, phase 2 study [44]. In total, 90 patients were enrolled in the study; 45 patients were diagnosed with osteosarcoma, and 42 of them were evaluable for efficacy. The 6-month ORR and 6-month non-progression were the primary end points for osteosarcoma. A PR was reported in 5 of 42 patients (12%), and 14 patients had 6-month non-progression. The median PFS and the median OS were 6.7 months and 10.6 months, respectively. Sixty-one of ninety patients included in the study experienced at least one serious adverse event. No drug-related deaths were reported. Cabozantinib could be a potential therapeutic option for advanced osteosarcoma, and requires additional research.

The role of **apatinib** in osteosarcoma management has not been explored extensively in prospective clinical trials; however, the results may encourage further investigation [45,46]. Xie L. et al. assessed apatinib (750 mg or 500 mg based on body surface area, once daily) in patients with progressive relapsed or unresectable high-grade osteosarcomas after failure of standard multimodal therapy, through an open-label phase 2 clinical trial [46]. The primary end point was determined as the ORR and PFS at 4 months. Thirty-seven patients were included in the analysis. The ORR and the 4-month PFS rate were 43.24% and 56.76%, respectively. The median PFS was 4.5 months, and the median OS was 9.87 months. Dose reductions or interruptions due to toxicity were reported in 25 of 37 (67.57%) patients, and no drug-related deaths were documented.

Different types of targeted therapies beyond TKIs have been investigated in clinical trials, including mTOR inhibitors, IGF-1R inhibitors, and PARP inhibitors, Aurora-A inhibitors, MEK inhibitors, CDK4/6 inhibitors, HER2 inhibitors, and EZH2 inhibitors [35]. Several mTOR inhibitors have been evaluated in clinical studies in osteosarcomas, including ridaforolimus [47], sirolimus [48], everolimus [38], and temsirolimus [49,50], either as a single-agent therapy or in combination with chemotherapy or other targeted therapies. However, the results regarding their efficacy are conflicting, and the use of mTOR inhibitors in clinical practice for osteosarcomas remains limited. Furthermore, clinical trials have investigated the clinical activity of IGF-1R inhibitors (robatumumab, RG1507, cixutumumab), with the results being discouraging in most of the studies [51,52,53]. Of note, the clinical evaluation of PARP inhibitors (olaparib) in osteosarcoma remains limited, and additional studies are required to reach a definitive conclusion [54]. Furthermore, alisertib, an Aurora-A inhibitor, has been tested in a phase 2 trial that enrolled 139 children and adolescents with recurrent/refractory solid tumors (including 10 with osteosarcoma) or leukemia. However, only 5 objective responses were reported (2 CRs and 3 PRs), but none were observed in patients with osteosarcoma [55]. Additionally, HER2 inhibitors such as trastuzumab failed to demonstrate clinical activity in osteosarcoma [56]. MEK inhibitors have been investigated extensively in pre-clinical models; however, the clinical evaluation is limited [35]. Currently, additional classes of targeted therapies, such as CDK4/6 inhibitors (NCT03242382, NCT03526250), and EZH2 inhibitors (NCT03213665), are under clinical investigation, enrolling patients with relapse or refractory advanced solid tumors including osteosarcoma.

## 4. Surgical Advances in the Management of Osteosarcoma

Whilst the judicious use of systemic oncological treatments has successfully improved the prognosis of patients with osteosarcoma, this therapy must be combined with appropriate local control in order to achieve a cure [57]. The surgical management of osteosarcoma will vary depending on the site and characteristics of the tumor, but the overall goal is to achieve complete resection with wide margins (R0). This involves removing the tumor as well as a layer of normal surrounding tissue. This is vitally important, as studies have shown an increased risk of local recurrence and decreased survival with positive or “marginal” margins [58]. Surgery can be offered to patients with localized osteosarcomas and to patients with metastatic disease, provided that all sites are resectable. Osteosarcoma can present in surgically challenging areas of the body, such as the pelvis, base of the skull, spine, and jaw, all of which may require specialist input from additional specialties such as neuro, spinal, general, ENT and maxillofacial surgeons.

Broadly speaking, the surgical treatment of osteosarcoma in the appendicular skeleton, where most osteosarcomas are found, falls into one of the following two categories: limb salvage (sparing) or sacrificing. Historically, amputations (limb sacrificing) formed the mainstay of surgical treatment; however, there has been a significant shift towards limb salvage options, without a detrimental effect on survival. This in part is due to the advancements in both surgical techniques and oncological therapies used in the adjuvant and neo-adjuvant setting. Studies have found an improvement in functional and psychological outcomes with the transition to limb salvage surgery, as well as a higher 5-year survival rate [59]. Developments in pre-operative (imaging) and intra-operative (surgical) techniques have allowed for better surgical planning, as well as more accurate tumor resections. Osteosarcoma tumor surgery is a delicate balance of achieving clear margins for prognostic benefit against the potential detrimental effects on function if too much tissue is resected. Surgical practices have progressed to optimize the oncological outcomes without forfeiting functional ones, provided that an attempt at limb salvage does not compromise adequate disease clearance [60].

### 4.1. Preoperative Imaging and Planning

As part of staging and oncological work up, a variety of different imaging modalities are used to characterize tumors. The current guidelines for osteosarcoma management recommend that each patient should have a plain radiograph, magnetic resonance imaging (MRI), computerized tomography (CT), and, in certain cases, a positron emission tomography (PET) combined with a CT scan. Where surgical planning is concerned, MRI scans have been shown to provide accurate depictions of tumor appearances, including their limits within and outside the bone, differentiating medullary disease involvement, as well as soft tissue involvement [60,61]. Resection margins can be accurately measured from high-resolution MRI scans, which can predictably inform the surgeon if a planned margin will be clear or affected by tumors. Studies have shown that MRI margin measurements with the closest predicted margin provided the smallest differences with pathology reports [62]. Although there is controversy in the literature with no defined ‘safe’ margin, it is generally accepted that a minimum soft tissue margin of >2 mm and a bony margin of >3 cm are required. The ‘barrier effects’ concept, introduced by Kawaguchi, further helps surgeon better plan and understand resection margins. This concept classified anatomical structures that provided resistance against tumor invasion (such as fascia, tendons, joint capsules, etc.) into thick (3–5 cm) and thin barriers (2 cm). Therefore, barrier effects can be considered to translate into distance equivalents; this means that at sites where barriers exist, surgeons can resect less of a margin than the true physical distance, allowing for more limb salvage options [57,63].

Intra-operative cancer detection techniques may pave the future for accurate tumor resection margins at the time of surgery. Currently, postoperative histopathology takes around two weeks to assess the success of surgery and validate the resection margins. Surgeons could benefit from a novel technique that would grant a rapid and objective determination of safe tumor margins at the time of tumor resection in theatre, in addition to classifying tumor types. Preliminary research with Raman spectroscopy has produced some promising results. When Raman spectroscopy is combined with principal component analysis (PCA) techniques, tumor types such as osteoblastic, chondroblastic, and telangiatatic osteosarcomas can be readily detected and identified in vitro [64].

### 4.2. Computer-Assisted Navigation

Given the complex anatomical nature of osteosarcoma resection, computer-guided technologies have been introduced to enhance intra-operative guidance in areas which require accurate osteotomies. Computer navigation incorporates all the imaging modalities used in preoperative planning, such as MRI and CT, in order to achieve a better balance between disease resection and preservation of disease-free tissue. The use of computer navigation has been particularly useful in resections of osteosarcoma from the pelvis and sacrum, as well as in difficult joint-preserving surgery [57,65]. The use of navigation devices has also been shown to potentially reduce the operating time and intra-operative blood loss. Joint-preserving surgery tries to offset the long-term failings of endoprosthesis, especially in skeletally immature patients; therefore, computerized assistance can have a role in aiding the preservation of the physis, whilst maintaining sufficient resection margins [60].

The current technology does have its limitations, and is not supported by robust literature; however, there are certainly grounds for future development. Robotic technology such as the MAKO robot (Stryker) has shown effective pedigree in arthroplasty techniques, with strong transferable principles to osteosarcoma resection. The robotic arm would allow cuts to be tracked in real-time, as well as aiding steadiness and maintenance of osteotomies in the desired plane, which should ultimately improve resection accuracy. Beyond this, technologies that involve augmented reality could be employed in the future to further build on the accuracy of tumor resection, whilst sparing important soft tissue structures [66].

### 4.3. Patient-Specific Instrumentation (PSI) and Three-Dimensional (3D) Printing

3D technology has revolutionized the surgical approach for osteosarcoma resections. It has aided surgeons to reconstruct and resect difficult tumor formations to preserve limb and function that would have otherwise been lost. 3D technology has both direct and indirect applications to help achieve this goal.

Indirectly, 3D-printed models of the tumor and anatomy can be used to better educate and inform patients pre-operatively in clinics, as well as help surgeons better visualize their approach and orientation before and during the procedure. Furthermore, 3D models can be used in the pre-operative planning stages, and in testing the appropriateness of implants used for reconstructions [60].

Directly, 3D technology has be used to design custom cutting templates and patient-specific instrumentation that is unique to the tumor characteristic for an individual patient. Studies have shown that PSI guides significantly improve resection accuracy as well as implant positioning. 3D printing has also enabled the use of a wider range of customizable implants, which are cheaper to produce and can be employed for reconstruction after resection. This is particularly relevant where the tumor resection involves complex anatomy, or is large in size and thus unsuitable for modular implant use. The use of custom implants offers clear advantages to both the surgeon and patient, especially when combined with computer navigation for precision resection. The concept of 3D printing in combination with computer navigation is being trailed in its early phase with the ‘just in time’ project by The Aikenhead Centre for Medical Discovery (ACMD), Australia [60,65].

The future of 3D printing may involve printed biodegradable implants as a drug delivery system. Although in its infancy, the concept is built on the principle of local delivery of pharmacological products (such as chemotherapy) or stem cells to promote implant osseointegration.

### 4.4. Reconstruction Options

Once the tumor is successfully excised, the surgeon’s attention turns to restoring function through effective reconstruction techniques that minimize immediate and long-term complications. A variety of reconstruction options exist, which are usually dictated by the tumor morphology and location. The options range from metallic gap spanning mega/endoprosthesis, forming the mainstay of treatment, to biological options that include autografts, allografts, and reimplantation of sterilized tumor bone. Each reconstruction method is associated with its own advantages and disadvantages.

Tumor endoprosthesis is the most commonly used technique in limb-sparing surgery. Many studies have shown reliable results with good functional outcomes and rapid restoration of weight-bearing status and mobility. Implant survival is estimated at 69–78% at 10 years, which is a significant improvement compared with previous implant designs [67]. Further advances in implant design and materials aim to offset complications that hinder implants, such as mechanical failure, loosening, and infection. Modern implants also allow for growth using non-invasive methods such as magnetic force. This becomes valuable as a tool for use in the skeletally immature population, who could be effected by issues in leg length discrepancy after tumor reaction from an affected growth plate [68].

Although less commonly performed, a variety of biological reconstruction options exist, and are usually limited to skeletally immature patients. These techniques include long segment allografts, inactivated reconstructions (reimplantation of sterilized tumor bone), allograft bone or inactivated bone combined with artificial joints, fibula transplantation, and bone transport techniques [69]. These techniques have traditionally been associated with slightly higher complication rates such as non-union, pathological fractures, and infections. With advancements in artificial prostheses, the role of biological reconstructions is likely to remain limited.

## 5. Conclusions

Osteosarcoma is a rare malignancy of mesenchymal origins, and its diagnosis depends on morphological characteristics rather than molecular characteristics. The multifaceted nature of osteosarcoma tumors poses a unique challenge for both surgeons and oncologists. The high risk of recurrence with inadequate margins, close proximity to critical structures, and large anatomical variances coupled with the lack of large-scale research, add to the complexity of osteosarcoma surgery. Advances in medical technologies, as well as an appreciation for tailored and individualized patient treatment, have allowed surgeons to vastly improve outcomes with a number of developments. On the other hand, prognoses for patients with metastatic disease remains dismal, and new systematic therapy approaches are being explored. Immunotherapy has been investigated without major breakthroughs being reported to date, whilst certain TKIs have demonstrated early clinical benefits that provide new therapeutic options in second-line treatments and beyond. Further studies are necessary to identify prognostic and predictive biomarkers in osteosarcomas.
